# Molecular detection and genetic characterization of *Mycoplasma gallisepticum* and *Mycoplasma synoviae* in selected chicken breeds in South Africa

**DOI:** 10.1186/s12879-024-09437-3

**Published:** 2024-06-05

**Authors:** Peter Ayodeji Idowu, Takalani J. Mpofu, Oliver T. Zishiri, Olusesan A. Adelabu, Khathutshelo A. Nephawe, Bohani Mtileni

**Affiliations:** 1 Department of Animal Sciences, Faculty of Science, Tshwane University of Technology, Private Bag X680, Pretoria, 0001 South Africa; 2 Discipline of Genetics, School of Life Sciences, University of KwaZulu-Natal, Private Bag X54001, Durban, 4000 South Africa; 3 Faculty of Health Sciences, Medical Microbiology Department, University of the Free State, PO Box 339, Bloemfontein, 9300 South Africa

**Keywords:** Mycoplasma *gallisepticum*, *Mycoplasma synoviae*, Poultry, qPCR, 16SrRNA, vlha gene, South Africa

## Abstract

**Background:**

The impact of chickens on maintaining the economy and livelihood of rural communities cannot be overemphasized. In recent years, mycoplasmosis has become one of the diseases that affect the success of South African chicken production. *Mycoplasma gallisepticum* (MG) and *Mycoplasma synoviae* (MS) are the most prevalent strains of Mycoplasma in South Africa. MG and MS are significant respiratory pathogens affecting the productivity of chickens. The present study aimed to molecularly detect using qPCR and characterize the presence of MG and MS using phylogenetic analysis. The phylogenetic analysis was utilized to clarify general evolutionary relationships between related taxa of different MG and MS observed in tracheal swabs from South African chicken breeds.

**Methods:**

Forty-five tracheal swabs of the Lohmann Brown (n = 9), Rhode Island Red (n = 9), Ovambo (n = 9), Venda (n = 9), and Potchefstroom Koekoek (n = 9) breeds were collected from symptomatic chickens present in the commercial farm. To detect MG and MS, DNA was extracted from tracheal swabs and faecal samples, and qPCR was performed with a 16 s rRNA (310 bp) and vlhA (400 bp) gene fragment. Following the sequencing of all the amplicons, MG, and MS dendrograms showing the evolutionary relationships among the five South African chicken breeds and the GeneBank reference population were constructed.

**Results:**

The qPCR revealed the presence of MG and MS in 22% (2/9) of the tracheal swab samples tested for MS only in Rhode Island Red breeds; 66.6% (6/9) and 33% (3/9) of the tested samples in Ovambo breeds; and 11.1% (1/9) and 44.4% (4/9) of the tested samples in Venda breeds. No MG or MS were detected in the Lohmann Brown or Potchefstroom Koekoek breed. Furthermore, qPCR revealed the presence of MG in pooled faecal samples from Lohmann Brown and Ovambo breeds. Eight different bacterial isolates were recognized from both samples. Four isolates were of the 16 s ribosomal ribonucleic acid (rRNA) gene (named PT/MG51/ck/00, PT/MG48/ck/00, PT/MG41/ck/00 and PT/MG71/ck/00) gene of *Mycoplasma gallisepticum*, and the other was *Mycoplasma Synoviae* variable lipoprotein hemagglutinin A (vlhA) gene (named PT/MSA22/ck/01, PT/MS41/ck/01, PT/MS74/ck/01 and PT/MS46/ck/01) which were available in GenBank. These isolates were successfully sequenced with 95–100% similarity to the isolates from the gene bank.

**Conclusion:**

The study revealed the presence of both MG and MS in the chicken breeds sampled. Furthermore, the different breeds of chicken were found to be susceptible to infection under the intensive or commercial management system. Therefore, continuous surveillance is encouraged to prevent the spread and outbreak of MG and MS in the poultry industry in South Africa.

## Background

It is well recognized that raising chickens in South Africa significantly improves rural livelihoods, promotes gender equality, and reduces food insecurity. Owing to its accessibility, affordability, and significant influence on economic growth and food security, chicken production has become a crucial and essential component of the nation's total agricultural earnings [1–4]. Nevertheless, chicken production successes are still hugely affected by disease prevalence in the commercial production sector, such as Mycoplasmas. According to Arroyo-Cruz [5], Mycoplasmas belong to the following taxa: phylum *Tenericutes,* class *Mollicutes*, order *Mycoplasmatales,* and family *Mycoplasmataceae.* They are spherical to filamentous cells with no cell wall around their cell membranes. They are phylogenetically related to gram-positive bacteria and are the tiniest and most basic self-replicating organisms currently known with a size ranging from 0.15 to 0.3 µm and a genome size of approximately 500 to 1000 genes [5, 6]*.* In chickens, mycoplasmas can lead to diseases such as chronic respiratory diseases of which this study only focuses on *Mycoplasma gallisepticum* and *Mycoplasma synoviae*. *Mycoplasma synoviae* causes synovitis majorly around the bursal and synovial membrane in chickens, which causes inflammation of the joints, tendons, and synovial membranes in chickens [6]. Common symptoms include breathing problems, nasal discharge, sinusitis, airsacculitis (inflamed mucous membrane in bird air sacs that becomes swollen and reddish), a decreased egg production, hatchability, and increased embryo mortality [6, 7]. *Mycoplasma gallisepticum* (MG) is an avian bacterial disease belonging to the class of Mollicutes (a bacterial class known to have no cell wall and a small genome size) and is a major agent of *avian mycoplasmosis,* such as *Gallus gallus* [8, 9]. It is known to be one of the most common avian mycoplasmas and has been associated with a reduction in feed conversion and egg production [10, 11]. Both Mycoplasma species are highly contagious irrespective of age and breed [7, 12], and are mostly attributed to respiratory tract difficulty such as sinusitis, respiratory abnormalities, or caseous, suppurative arthritis in the hock and foot joints, in chickens [13]. Due to its vertical nature (egg production), horizontal spread (drinking, feeding, and other daily activities), and relatively small size, its eradication and prevention are complex in poultry-infected farms [13, 14]. Ishfaq et al. [12] observed that MG infection accounted for up to 30% reduction in weight gain, a 10–20% reduction in egg production, and approximately 20% reduction in the feed conversion ratio. Several authors have stated that both MG and MS are pathogenic mycoplasma species that infect poultry and result in significant financial losses [10, 14, 15]. Interactions between Mycoplasma species and other major infected epithelial cells play important roles in the early stages of various inflammatory processes. These activities include the secretion of inflammatory cytokines and chemokines, the recruitment and activation of immune cells and the activation of the pattern recognition receptor [16]. Understanding the prevalence and molecular characterization of MG and MS in South African chicken breeds is crucial for effective disease management and the implementation of appropriate control strategies. Previous studies have reported MG outbreaks in backyards and commercial poultry farms in other developed countries, but little is known about the occurrence and diversity of MG in different breeds of South African chickens. Therefore, to understand the presence and abundance of bacterial prevalence in the chicken gastrointestinal tract, swab samples such as tracheal swab and faecal samples can also be used [17]. Previous studies have used culturing, ELISA, and qPCR methods for mycoplasma detection [18, 19]. Nevertheless, qPCR is one of the best detection methods because of its high level of reliability and speed [20, 21]. These techniques can help individuals identify different strains of MG and MS and provide valuable information on the genetic diversity and potential transmission routes of the pathogen in chickens [22]. Due to the financial implication of this procedure, only a small number of chicken farmers constantly detect the prevalence of MG and MS in the flock. This has led to cases of reduced egg production, sunken eye, and in extreme cases premature death [10, 11, 13].

Therefore, this study aimed to investigate the presence and genetic diversity of MG and MS in different chicken breeds in South Africa using qPCR techniques. This research will contribute to our understanding of the epidemiology of MG and MS in South African chicken breeds and to suggest control measure of its prevalence in chicken production. Overall, this study provides important information on the occurrence, genetic diversity, and possibility of variation in MG and MS in chicken breeds in South Africa.

## Methods

### Specimen collection and ethical clearance

Tracheal swab, faecal swab and pooled faecal samples were collected from forty-five (45) randomly symptomatic South African chicken breeds: Lohmann Brown (hybrid) n = 9, Rhode Island Red (hybrid) n = 9, Ovambo (indigenous) n = 9, Venda (indigenous) n = 9, and Potchefstroom Koekoek (indigenous) breeds n = 9 under intensive poultry system. For this study, chicken breeds at 25 weeks and 35 weeks of age showing clinical signs of respiratory symptoms such as nasal discharge, sunken eye, and sneezing, and other symptoms like reduced egg production, painful joints were used for this study. All the birds sampled are in the same pen and were live chickens.

The farm is in Newcastle, KwaZulu Natal Province in South Africa. The poultry farm is registered with the South African Poultry Association with strict biosecurity measures such as dipping at entrance, limiting visitors, clean water, and the supply of farm clothing at entrance, among others. The breeds are raised primarily for sales to generate income and are the predominant breeds of chickens in South Africa chicken production. The chickens were arranged in individual pen based on phenotypic characterisation (breed type). All the chicken breeds were vaccinated at six weeks for *Mycoplasma* vaccination. Tracheal swab collected from each pen was placed in a sterile tube, while the faecal samples were collected from the cloacal region of the same chicken and placed in an EDTA tube with a DNA shield.

Before the commencement of the research, the study was approved by the Animal Research Ethics Committee of the Faculty of Science, Tshwane University of Technology AREC2021/10/002. Also, the study is in accordance with Animal Research Reporting of In Vivo Experiments (ARRIVE) guidelines (https://arriveguidelines.org). Written informed consent was obtained from the farm owner before sample collection. Tracheal swabs and faecal samples were collected once and subjected to qPCR test to determine the presence and abundance of MG and MS. The swab samples were subsequently stored at -80 °C until further analysis. Previously, the samples were stored at -20 °C. The tracheal swabs were collected and extracted from the chickens individually.

### DNA Extraction from Tracheal Swab

For samples prepared according to the DNA / RNA ShieldTM specifications, the faecal sample was homogenized by bead bashing or other homogenization protocols. The tracheal swabs collected were eluted in 2 mL of sterile PBS (phosphate-buffered saline). Mycoplasma DNA was extracted from a 1 ml aliquot of stationary-phase culture using the Genelute Genomic DNA Kit according to the manufacturer’s instructions (Sigma). DNA was extracted from the swabs by swirling the swabs in 1 ml of PBS, removing the swabs, and then using a GenElute Kit as described above. DNA was extracted from the faecal samples by removing 1 cm of 2 portions of faecal using sterile instruments, after which the samples were placed in 1 ml of PBS. The DNA was homogenized to produce a suspension, after which the DNA was extracted using a Sigma‒Aldrich Tissue Kit according to the manufacturer’s instructions.

### DNA Extraction of Faecal Swab

A mixture of 95 μl of water, 95 μl of solid tissue buffer (blue), and 10 μl of proteinase K was added to the pooled faecal sample (≤ 25 mg) in a microcentrifuge tube. The mixture was then vortexed for 10 s, after which the tube was incubated at 55 °C until the tissue was solubilized (range of 1–3 h). The device was well combined before moving forward. Subsequently, 400 μl of Genomic Binding Buffer was mixed with 200 μl of supernatant and vortexed for 10–15 s. After that, the mixture was sent in a collection tube to a Zymo-SpinTM IIC-XLR column. After 60 s, the mixture was centrifuged at ≥ 12,000 × g. The collection tube and flowthrough were subsequently discarded, after which a fresh collection tube containing 400 μl of DNA prewash buffer was added to the spin column. Subsequently, the collection tube was emptied and centrifuged for one minute at a force of at least 12,000 × g. The spin column was filled with 700 μl of g-DNA wash buffer. After one minute of centrifuging at ≥ 12,000 × g, the collection tube was emptied. Afterward, the spin column was supplemented with 200 μl of gDNA wash buffer. After centrifuging for one minute at a rate of ≥ 12,000 × g, the collecting tube containing the flowthrough was removed. Subsequently, the spin column was moved to a sterile microcentrifuge tube, and the matrix was immediately covered with ≥ 50 μl of DNA elution buffer. After the mixture was incubated for five minutes at room temperature, the DNA was extracted by centrifugation for one minute at maximum speed. The DNA that was eluted was then stored at ≤ -20 °C for further analysis.

### Detection of *Mycoplasma gallisepticum*

The presence of MG was investigated using qPCR targeting a 310 bp fragment of the proline-rich domain of the 16 s rRNA adhesin-encoding gene [23]. The forward primer 16 s rRNA-F (5' AGCTAATCTGTAAAGTTGGTC’) and the reverse primer 16 s rRNA-R (5’ GCTTCCTTGCGGTTAGCAAC 3’) were used to type the DNA samples [23]. Amplification of the V3 region of the 16S rRNA gene was performed according to the methods of Muyzer et al. (1993). Amplification of the with minor modifications using the universal template to 49 μl of a reaction mixture containing 10 mM Tris/HCl (pH 9.0), 1.5 mM MgCl_2_, 50 mM KCl, 0.1% Triton X-100, 0.2 mM of each deoxynucleotide triphosphate and 0.5 U of Taqgold (Applied Biosystems). The cycling procedure involved initial denaturation at 94^0^ C for 5 min; 35 cycles of 94 ^0^C for 5 min, 50 ^0^C for 30 s, and 68 ^0^C for 1 min; and final elongation at 68 °C for 10 min. The samples were kept at 4 ^0^C until analysis. The integrity of the qPCR amplicons was visualized on a 1% agarose gel (CSL-AG500, Cleaver Scientific Ltd.) stained with EZ-vision® Bluelight DNA Dye.

### Detection of* Mycoplasma synoviae*

For Mycoplasma synoviae, a qPCR test was run that specifically targeted a 400 bp region of the vlhA gene, which encodes a plentiful immunodominant surface protein. Proline-rich repeat (PRR) tandem repeats and the highly polymorphic RIII region were present in the target region, enabling subtyping and typing of a strain of MS [24]. The forward primer vlhA-F (5'TACTATTAGCAGCTAGTGC 3’) and the reverse primer vlhA-R (5’AGTAACCGATCCGCTTAAT 3’) were used. A previously characterized MS sample was included as a positive control in all the qPCRs. The vlhA-PCR mix was carried out in a total volume of 25 μL per sample, comprising 0.25 μL of Taq DNA polymerase (5U per μL), 2.5 μL of 10X PCR buffer, 0.5 μL of 50 mM MgCl2, 0.5 μL of 10 mM deoxynucleotide triphosphates (dNTPs), and 1 μL of each primer. Consequently, 2 μL of extracted DNA was added as a template, along with 17.25 µL of deionized distilled water. In an Eppendorff thermal cycler (Eppendorff, Hamburg, Germany), the vlhA-PCR reaction was carried out as follows: 94 ˚C for 5 min, then 35 cycles of 60 s at 94 ˚C, 60 s at 53 ˚C and 1 min at 72 ˚C, with a final extension cycle of 10 min at 72 ˚C. Amplified products were stained using ethidium bromide (0.5 µg per mL) and subjected to agarose gel electrophoresis.

### Sequencing and phylogenetic analysis

**Fig. 1:**
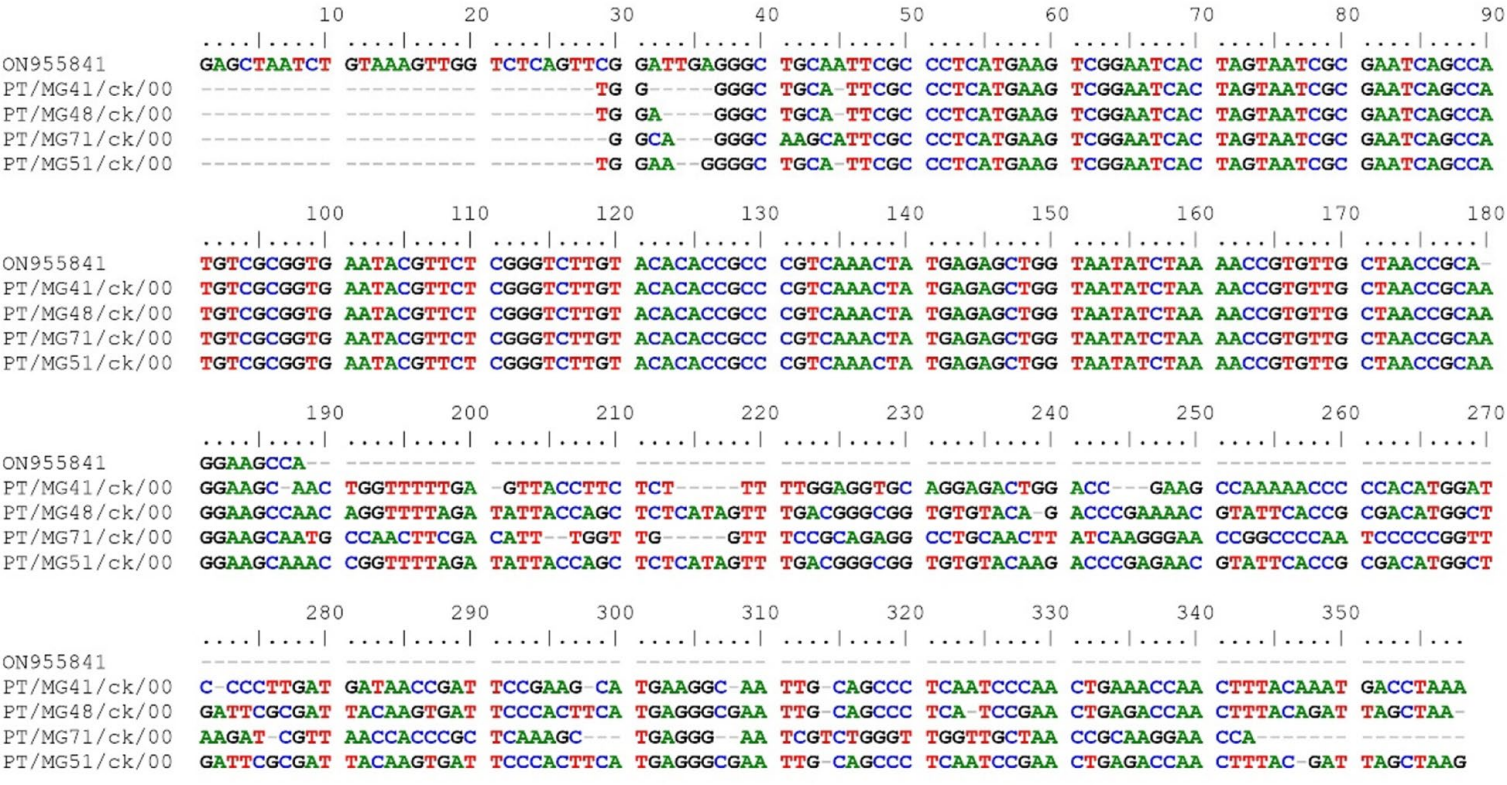
Nucleotide sequences of the 16S rRNA gene of MG strains detected in the present study and the reference strain ON955841

Using a commercial sequencing service (Inqaba Biotec, RSA), the vlhA MS and 16S rRNA MG amplicons were sequenced both ways. The presence of MG was verified by the visualization of 237–310 bp amplicons. With the use of BioEdit software, the acquired sequences were modified and assembled. The nucleotide BLAST technique with the GenBank database (www.ncbi.nlm.nih.gov/BLAST) was used to determine the nucleotide identity of the vlhA sequences. For Molecular Evolutionary Genetics Analysis (MEGA X), the neighbour-joining method was used with the maximum likelihood model to create dendrograms [25]. Based on 1,000 replicates, bootstrap values were computed, and values greater than 70 were deemed significant. Phylogenetic analysis of previously published strains and published matching sequences of 25 MS and MG reference strains were performed in addition to the sequences obtained in this study (Fig. 3 and 4). Only MG samples for which only two partial sequences of two genes were successfully sequenced and were used to construct the dendrogram for MG sequences. The MG sequences identified via 16S rRNA analysis were compared with those of published strains, including reference strains (Fig. 1).

## Results

The result of the tracheal swab collected from symptomatic chicken breeds in this study are as follows: none of the (0.00%) Potchefstroom Koekoek chicken breeds were positive for MG and MS. However, Rhode Island Red breed, 0 (0.00%) and 2 (22.22%) were tested positive for MG and MS Also, the LB breeds, 0 (0.00%) and 3 (33.33%) were tested positive to MG and MS. Furthermore, six (66.66%) and three (33.3%) Ovambo breeds were positive for MG and MS. Lastly, one (11.11%) and four (44.44%) Venda breed samples were positive for MG and MS using a qPCR assay and it was performed with a 16 s rRNA (310 bp) gene fragment for MG and vlhA (400 bp) gene fragment MS (Table 1). In the same study, the pooled faecal sample revealed that MG was abundance in Lohmann Brown and Ovambo breeds only, these samples were collected from the same chicken breeds (Table 1).

**Table 1:** Detection of *M. gallisepticum* in faecal samples and tracheal swabs sampled from different chicken breeds in South Africa

Breeds	Age	Total examined Samples	*Mycoplasma synoviae* Positive Samples		*Mycoplasma gallisepticum* Positive samples	
			Faecal (Pooled)	Swab	Faecal (Pooled)	Swab
Rhode Island Red	35 weeks	9	-	+ (2)	-	-
Lohmann Brown	35 weeks	9	-	+ (3)	+ (1)	-
Ovambo	25 weeks	9	-	+ (3)	+ (1)	+ (6)
Venda	25 weeks	9	-	+ (4)	-	+ (1)
Potchefstroom Koekoek	25 weeks	9	-	-	-	-

### MG gene: 16S rRNA; MS gene: VlhA

Supplementary Table 3 contains all MS strain used for this study with their respective country and accession numbers.

The nucleotide sequences of this study were compared with the GenBank sequences with respective nucleotides and percentages of similarity ranging from 95%-100% (Supplementary Table 1). For the 16 s rRNA MG sequence analysis, all the vlhA amplicons were sequenced successfully, and the MS strains were PT/MG51/ck/00, PT/MG48/ck/00, PT/MG41/ck/00, PT/MG71/ck/00, PT/MSA22/ck/01, PT/MS41/ck/01, PT/MS46/ck/01, PT/MS74/ck/01 and PT/MS78/ck/01 (Supplementary Table 3). Sample PT/MG48/ck/00 showed a percentage of nucleotide similarity of 96% with the ON955841 Indian strain, 96% with the OR388785 Pakistan strain and 96% with the South African strain (MH539140) in 16S rRNA (Supplementary Table 1). The sample PT/MG71/ck/00 showed a percentage of nucleotide similarity of 97% with MH876250 Indian strain, 98% with MN069583 Indian strain, 98% with USA strain NR104952 and 97% with MW517331 Indian strain (Supplementary Table 2). The sample PT/MG51/ck/00 showed 97% nucleotide similarity with the MZ373235 Pakistan strain, 97% nucleotide similarity to the ON955841 Indian strain and 97% nucleotide similarity with the OR346328 Pakistani strain (Supplementary Table 2). PT/MS46/ck/00 showed a percentage of nucleotide similarity of 100% with the MH679841 Hungarian strain, the MF737477 Polish strain and the ON191514 Thai strain in the vlhA gene. PT/MS78/ck/00 exhibited 100% nucleotide similarity with the AB501271 Japanese strain, 100% similarity with the AF464937 Australian strain, 98% similarity with the Brazilian strain KC506905 (Supplementary Table 3). PT/MSA22/ck/00 showed 99% nucleotide similarity with the KC832812 Israeli strain, 98% nucleotide similarity with the KC506807 Hungarian strain and 95% nucleotide similarity with the MH679904 Brazilian strain. PT/MS41/ck/00 exhibited 96% nucleotide similarity with the MH679904 Hungarian strain, 99% nucleotide similarity with the KC832812 Israeli strain and 99% nucleotide similarity with the MH679840 Hungarian strain (Supplementary Table 1).

Multiple sequence alignments of *Mycoplasma gallisepticum* from this study and the reference strain are shown in Fig. 1. PT/MG41/ck/00, PT/MG48/ck/00, PT/MG71/ck/00 and PT/MG51/ck/00 had the following base pairs: 180, 196, 193 and 199, respectively (Fig. 1).

**Fig. 2:**
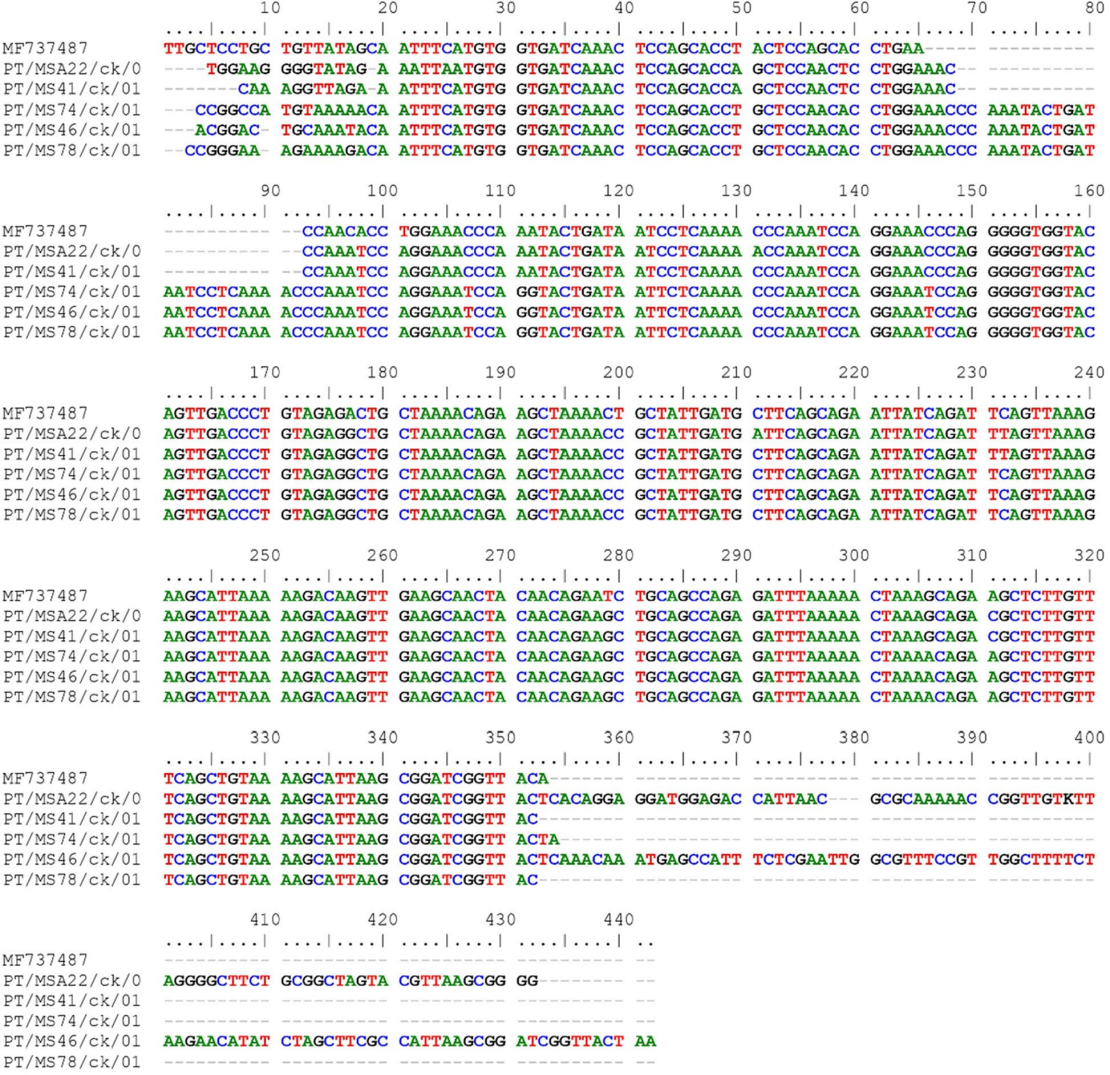
Nucleotide sequences of the vlhA gene of MS strains detected in the present study and the reference strain MP737487

Multiple sequence alignment of *Mycoplasma synoviae* from this study and the reference strain. PT/MSA22/ck/00, PT/MS41/ck/00, PT/MS74/ck/00, PT/MS46/ck/00 and PT/MS78/ck/00 have the following base pairs: 139 bp, 127 bp, 149 bp, 150 bp and 149 bp, respectively. According to the MS sequence analysis, the *vlhA* amplicons were successfully sequenced. The MS strains analysed were previously described (Fig. 2).

Supplementary Table 2 contains all the strains of MG used for this study with their respective country and accession numbers.

**Fig. 3:**
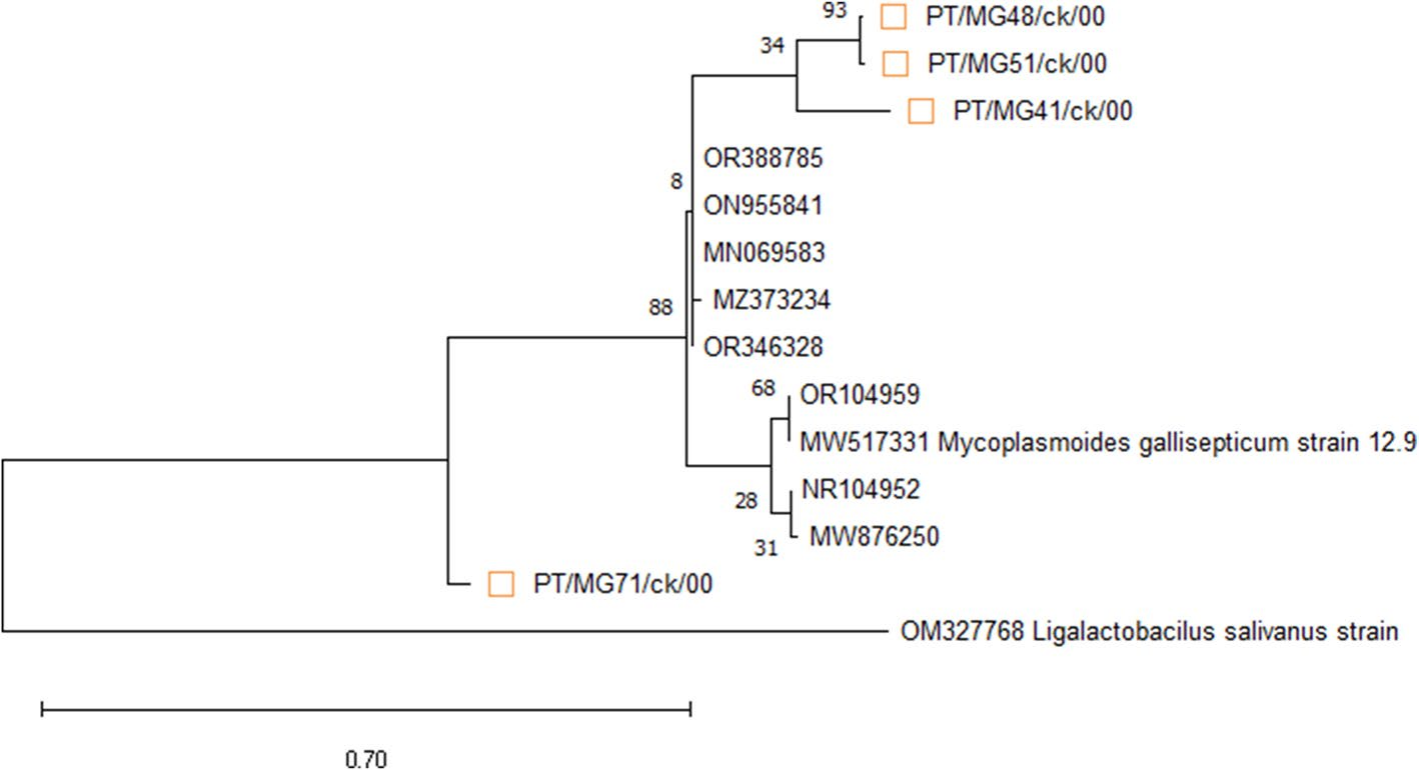
Phylogenetic tree based on the alignment of the nucleotide sequences of the 16S rRNA genes of the MG accession number (strain on supplementary Table 2) detected in the present study (square) and reference strains. Only bootstrap values > 70 is reported

The dendrogram obtained from this study revealed that the MG strains belonged to four different subclusters (Fig. 3). PT/MG41/ck/00 clustered with OR104959, while PT/MG48/ck/00 was closely related to OR346328. PT/MG51/ck/00 formed three separate strains from the reference strains MN069583, ON95584 and MW517331.

Supplementary Table 3 contains all the strains of MS used for this study with their respective country and accession numbers.

The dendrogram obtained from this study revealed that the MS strains belonged to four different subclusters (Fig. 3). PT/MS78/ck/01 has similar nodes to ON191527, while PT/MS41/ck/01 has similar nodes to KC832812. PT/MS46/ck/01 formed a separate strain from the reference strain ON191527.

**Fig. 4:**
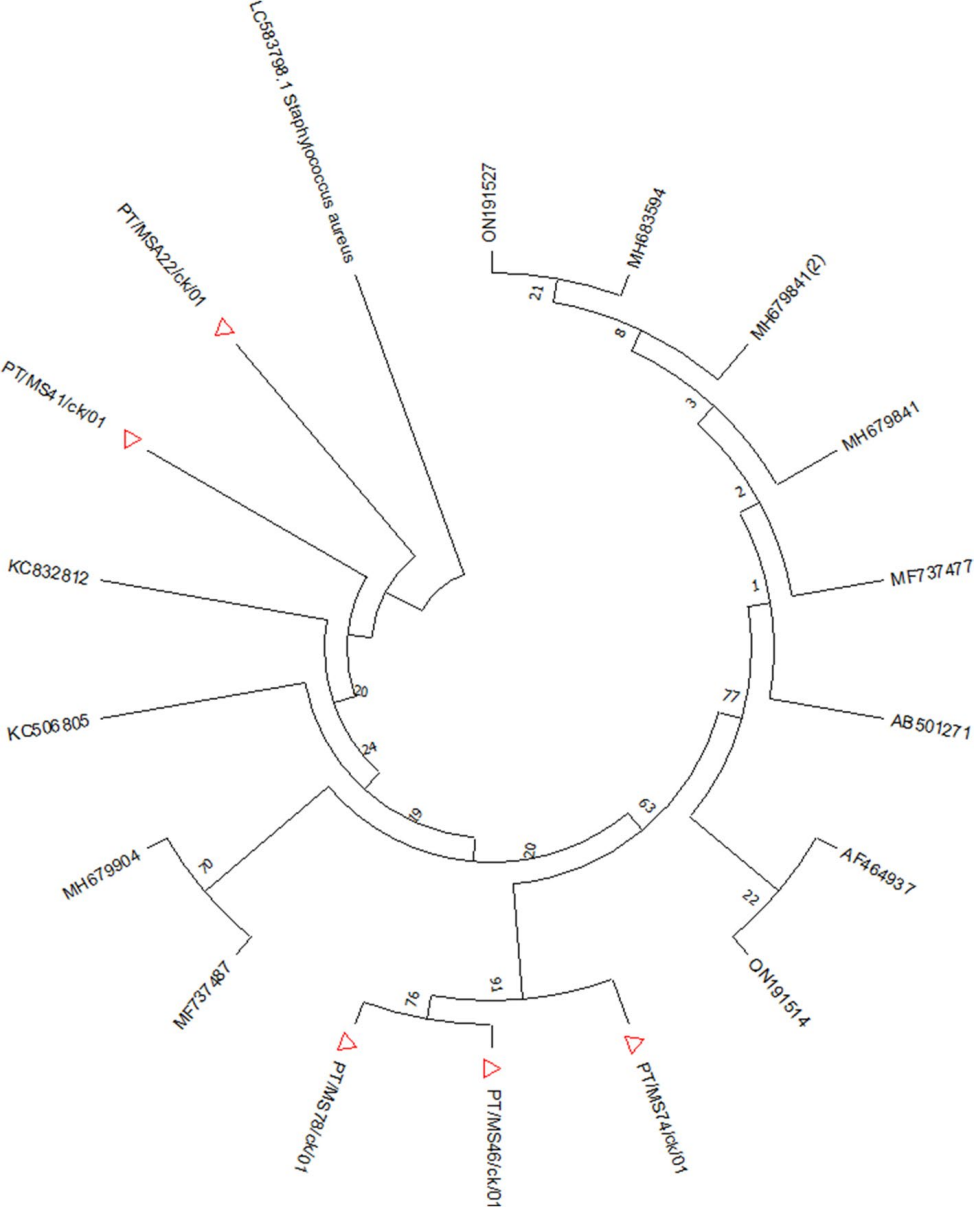
Phylogenetic tree based on the alignment of nucleotide sequences of *vlhA* genes of MS accession numbers (strain on supplementary Table 3) detected in the present study (triangle) and reference strains. Only bootstrap values > 70 is reported

The PT/MS41/CK/01 strain shares 84% similarity with the KC832812 Israeli strain of MS. Additionally, there was a cluster of PT/MS74/CK/01 strains with Japanese MS strains with 74% similarity. Finally, there was a cluster between PT/MS78/CK/01 and the MS strain Thailand, with reference number ON191527 (Fig. 4). The two strains are closely related, showing a high level of similarity from common ancestors. Nevertheless, PT/MS46/CK/01 underwent speciation from two of the above strains (ON191527 and PT/MS78/CK/01), which were from the same ancestor but not closely related. The dendrogram obtained from this study revealed that the MS strains belonged to four different subclusters (Fig. 4). PT/MS41/ck/00 and PT/MSA22/ck/00 clustered with the KC832812 (Israel) strain at 84% and 81%, respectively. The South African strains exhibited a strong evolutionary trend compared with the Israeli strains. Additionally, the ON191527 (Thailand) strain has a strong evolutionary relationship with two South African strains from this study, PT/MS78/ck/00 and PT/MS46/ck/00, with 79% and 78%, respectively, similarity. Interestingly, PT/MS74/ck/00 is evolutionarily distant from the other four strains observed in this study (Fig. 4). This strain could be genetically diversified from the other three strains reported in this study.

## Discussion

It is important to note that poultry production is a viable and ever-growing business among other agricultural-related businesses in South Africa, as it accounts for the most affordable protein sources [3]. Recently, there have been cases of sunken eyes, breathing difficulties, reduced egg quality, and decreased egg production in the sampled chicken breeds which are all symptoms related to MG and MS [19, 26, 27]. The objective of the present study was to molecularly detect the presence and genetically characterize MG and MS in different chicken breeds across South Africa using qPCR techniques.

This study revealed the presence of MG in swab samples in all symptomatic chicken breeds, except Potchefstroom Koekoek. Of the 45 samples that showed symptoms, only 12 samples (26.66%) and 7 samples (15.55%) of the 45 samples were positive for MS and MG. Our findings demonstrated that the prevalence of MG and MS in commercial chicken farms. Chandhar et al. (2018) reported cases of 13.33%, which is similar to the findings of this study (26.66%) This similarity could be due to similar smaller sample size and similar chicken farm management (intensive or commercial). However, the study by Felice et al. [28] on a chicken backyard farming system in Italy recorded a relatively higher prevalence of MG and MS. The differences observed may be due to larger sample size, less or no vaccination programs, no restrictions in spread of diseases, and backyard production system (which allows chickens to roam around). This current study observed a low (15.55%) level of prevalence of MS, which is dissimilar to the study by Khalifa et al. [29] who reported cases of 71.6%. This could be attributed to the farming system (local farms) where samples were collected. Khalifa et al. [29] reported that MG and MS can easily be transmitted across poultry pens, supporting the current finding that MG and MS are present in both tracheal swabs and faecal samples. This may suggest that there is horizontal transmission of mycoplasma within poultry chickens.

Phylogenetic analysis of the MG 16S rRNA gene sequences revealed a high percentage of nucleotide similarity. This finding corresponds to the reported sequences of MG strains reported in Brazil, India, Pakistan, and the United States [13]. Since the 16S rRNA gene is a highly conserved gene and ubiquitous in bacteria and archae, the similarity in this region is high with less variation. Previous research has used this gene region to identify a wide diversity of microbes within a single sample and single workflow and identification of taxa present in the sample [26, 27, 30, 31]. This study observed an evolutionary relationship among all isolates. Two strains were observed in this study, namely, PT/MG71/ck/00 (99%) and PT/MG41/ck/00 (98%), which had high similarity to those in Brazil, but only with accession number OR104959. This finding showed a high level of similarity between these two isolates. Furthermore, PT/MG48/ck/00 and PT/MG51/ck/00 from this study have high levels of similarity with those from a study from India, with accession number ON955841. Furthermore, the current study showed a high level of evolutionary relationship with similar studies in Hungary, Poland, Thailand, Japan, and Egypt [28, 32]. This high level of similarities shows that the MG isolates are genetically related to the isolates of this study. Additionally, IT/MG41/ck/00, IT/MG48/ck/00, and IT/MG51/ck/00 formed a separate cluster from all other MG strains analysed, indicating a high degree of similarity (99.6%). However, IT/MG71/ck/00 was not closely related to the other three strains in this study. This could suggest the presence of a new strain among the other strains detected in South Africa chicken production.

Recently, several studies have conducted epidemiological research and MS strain studies using the single copy conserved region of the MS vlhA gene [7]. Due to the ability of the vlhA gene to encode surface lipoproteins, which play a crucial role in virulence and immune evasion. Understanding the prevalence of this gene and its genetic characterisation in South African chicken breeds will be a great necessity in effective control measures. A dendrogram of the *vlha* sequences of the MS strains detected in this study shows that the PT / MSA22 / CK / 01 study was clustered with Israel KC832812 and Hungary KC506807, with percentage similarities of 99 and 98%, respectively. Together with the previously documented Thailand isolate ON191527 formed the same clade with PT/MS78/CK/01 formed a distinct clade named PT/MS46/CK/01. Furthermore, the previously documented Israel isolate KC832812 formed the same clade with PT/MS41/CK/01 and a distinct new clade named PT / MS21 / CK / 01 This implies that this study has produced important findings about the distribution and diversity of MS in South African chicken breeds.

Despite all attempts to produce mycoplasma-free poultry in South Africa poultry production, the prevalence of MG and MS across poultry houses corroborates that MG and MS can be transmitted horizontally through the possible inhalation of contaminated droplets of air in feed [33]. Furthermore, MG and MS were detected from both swab and faecal samples, this was done to investigate the extent of abundance of MG and MS from the symptomatic chicken breeds. It is important to note that the swab is the most preferred sample for mycoplasma detection studies as bacteria are most seen in the gastrointestinal tract [34]. Additionally, air easily contributes to the transmission of bacterial pathogens to the tracheal region faster than to the lower part of the body [33]. Therefore, conscious efforts should be made to ensure strict adherence to biosecurity measures and early detection in commercial chicken farm Also, selection of chicken breeds with higher humoral responses to mycoplasma, use of essential oils [26], use of herbal extracts [35], use of phytogenic products [36], use of prebiotics and antibiotics [37], use of probiotics [14], use of synbiotics [38], use of biologically sensitized nanoparticles [39] and possibly chickens with high levels of mannose-binding lectin [40] in chicken production should be encouraged.

## Conclusion

The findings of this study show the presence and genetic characterisation of MG and MS strains in South African chicken breeds. Four strains of MG and MS genotypes have been found in South Africa using the 16 s rRNA and vlhA gene, and some of them seem to differ genetically from the strains reported from previous studies. Periodic surveillance should be carried out to improve vaccination programs and reduce financial losses that could be related to the effect of MG and MS infections. Further research should be conducted using another gene of interest, in vivo pathogenicity, and antimicrobial resistance of MG and MS in South African chicken breeds present in commercial chicken farms.

## Data Availability

The datasets used and/or analysed during the current study are available at NCBI database with the ascension number PP343275, PP343276, PP343277 and PP343278 for Mycoplasma gallisepticum. While ascension numbers PP789056, PP789057, PP789058 and PP789059 for Mycoplasma synoviae.

## Abbreviations

MG: *Mycoplasma* *gallisepticum*
MS: *Mycoplasma* *synoviae*
vlhA: Variable lipoprotein hemagglutinin genes
rRNA: Ribosomal RNA
PRR: Proline-rich repeat
qPCR: Quantitative Polymerase Chain Reaction
bp: Base pair
MEGA: Molecular Evolutionary Genetics Analysis
ARRIVE: Animal Research Reporting of In Vivo Experiments
